# Novel Texture Feature Descriptors Based on Multi-Fractal Analysis and LBP for Classifying Breast Density in Mammograms

**DOI:** 10.3390/jimaging7100205

**Published:** 2021-10-06

**Authors:** Haipeng Li, Ramakrishnan Mukundan, Shelley Boyd

**Affiliations:** 1 Department of Computer Science and Software Engineering, University of Canterbury, Christchurch 8140, New Zealand; mukundan@canterbury.ac.nz; 2 Canterbury Breastcare, St. George’s Medical Centre, Christchurch 8014, New Zealand; shelley.boyd@pacificradiology.com

**Keywords:** breast density classification, local binary patterns, multi-fractal analysis, texture feature, mammogram

## Abstract

This paper investigates the usefulness of multi-fractal analysis and local binary patterns (LBP) as texture descriptors for classifying mammogram images into different breast density categories. Multi-fractal analysis is also used in the pre-processing step to segment the region of interest (ROI). We use four multi-fractal measures and the LBP method to extract texture features, and to compare their classification performance in experiments. In addition, a feature descriptor combining multi-fractal features and multi-resolution LBP (MLBP) features is proposed and evaluated in this study to improve classification accuracy. An autoencoder network and principal component analysis (PCA) are used for reducing feature redundancy in the classification model. A full field digital mammogram (FFDM) dataset, INBreast, which contains 409 mammogram images, is used in our experiment. BI-RADS density labels given by radiologists are used as the ground truth to evaluate the classification results using the proposed methods. Experimental results show that the proposed feature descriptor based on multi-fractal features and LBP result in higher classification accuracy than using individual texture feature sets.

## 1. Introduction

Breast density is a critical bio-marker which indicates the possibility of developing breast cancer in the future for women. High breast density is caused by a high percentage of fibro-glandular tissue and reduces the effectiveness of mammography screening [1]. Related research work shows that women with extremely dense breast could suffer four to six-fold higher risk of developing breast cancer than other females with low breast density [2]. Dense tissue areas in mammograms also cause the ‘masking’ effect leading to reduced sensitivity when radiologists visually assess breast lesions or early signs of cancer, such as lumps and calcification clusters [3]. Breast density is gaining significant attention because it is closely associated with higher cancer risk, increased incidence of interval cancer and reduced mammographic sensitivity.

Breast density measurements help identify and target women who could benefit from tailored screening options such as increased (or decreased) screening interval or supplemental screening via alternative modalities [4]. Breast density categories currently in use are six-class-categories (SCC) [2], Wolfe’s four categories [5], and the Breast Imaging-Reporting and Data System (BI-RADS) [6]. BI-RADS criterion proposed by the American College of Radiology (ACR) has been widely used in clinical applications and includes four density categories: fatty, scattered density, heterogeneously dense, and extremely dense. A simplified two-category model (“fatty and sparsely dense”, “heterogeneously and extremely dense”) is also sometimes used [7]. It may be noted here that the robustness of clinical workflows that heavily depend on radiologists’ subjective visual assessments may be affected by inter- and intra-observer disagreement [8].

The development of computerised mammogram interpretation methods is still an active research field. Advanced machine learning and image analysis algorithms are currently being developed to extract diagnostically relevant features and derive quantitative measurements for improving radiologists’ workflow [9]. For breast density classification in mammograms, extracting effective features plays an important role in obtaining accurate classification results. A number of feature extraction methods have been proposed and studied in literature, with the aim of capturing higher order statistical information and improving the classification accuracy.

Different image characteristics have been considered and used as the feature descriptors, including image intensity, texture patterns, morphological features, and statistical information. Mario et al. [7] extracted multiple features based on image intensity, histograms, and gray level co-occurrence matrix (GLCM) to classify mammograms. Wrappers were used for feature selection and improving the classification performance. Oliver et al. [10] combined intensity, texture, and morphological features to classify pixels in mammograms into two categories (fatty and dense) using an SVM classifier. Statistical features including mean, standard deviation, smoothness, third moment, uniformity, and entropy were used to classify mammograms into three density categories in [11]. Qu et al. [12] proposed a fuzzy-rough refined image processing method to enhance local image regions and extract GLCM based statistical features for classifying mammographic density. The method in [13] extracted 137 pixel-level features containing intensity, GLCM, and morphological features, to group pixels into fatty or dense classes. The work in [14] extracted 21 features based on intensity and fractal texture features, and SVM was used to classify the Mammographic Image Analysis Society (MIAS) mammograms into three categories. Muhimmah et al. [15] used multi-resolution histogram method to analyse texture features, and mammograms were classified to three density categories by a directed acyclic graph (DAG)-SVM classifier. Chen et al. [16] investigated five texture feature sets separately, including LBP, local grey level appearance (LGA), textons (MR8 texton and image-patch texton), and basic image features (BIF). Their experimental results showed that image-patch texton features produced a higher classification accuracy for 4 BI-RADS categories classification. A multi-scale blob detection method was proposed in [17] to recognize the fatty and dense tissue present in mammograms. This method was used to analyse MIAS mammograms and experimental results revealed some initial relations between the BI-RADS density category and the average relative tissue (fatty and dense) area in mammograms. Zheng et al. [18] used a lattice-based approach to extract statistical and structural features for analysing parenchymal texture in mammograms.

Recently, deep learning-based methods have been used to analyse mammograms for evaluating and classifying breast density, with promising results. Mohamed et al. [19] proposed a CNN model based on AlexNet to distinguish between two BI-RADS categories (‘scattered density’ and ‘heterogeneously dense’) which often lead to disagreements in radiology assessments [20]. A CNN architecture was designed in [21] to learn the features from a multitude of sub-images and to classify them into dense and fatty tissues. Lee et al. [22] used fully convolutional network (FCN) to segment breast regions and fibro-glandular areas with the aim of estimating percentage density. In [13] a deep convolutional neural network (DCNN) was trained to classify mammographic pixels into fatty or dense class. A probability map of breast density (PMD) was generated and used to estimate percentage density. Kallenberg et al. [1] proposed an unsupervised deep learning model to segment dense breast region from mammograms. However, applying deep learning methods to classify breast density requires a huge number of training images with accurate annotations by clinicians [23], and some datasets used in related work are not publicly available.

Texture analysis methods, particularly using descriptors such as LBP and its variants, have been shown to be useful in extracting relevant image structure information for classification tasks. Ojala et al. [24] proposed a method using local binary patterns (LBP) to describe image texture patterns. Due to its simplicity and efficiency, LBP has been studied widely and several variants were proposed for extracting texture features and classifying medical images. In [25], LBP was extended to elliptical LBP (ELBP), and mean-elliptical LBP (M-ELBP) by considering various neighbourhood topologies and different local region scales. It has been reported that M-ELBP gave a satisfactory classification result (77.38 ± 1.06) on MIAS dataset. Tan et al. [26] proposed local ternary patterns (LTP) using a three-value encoding approach compared to two-value encoding of LBP. Rampun et al. [27] extracted LTP based texture features to classify MIAS mammograms into four BI-RADS categories. Nanni et al. [28] proposed local quinary patterns (LQP) by extending LBP from a binary encoding system to a five-value encoding system, and used three different medical image classification tasks to test this new texture descriptor. Subsequently LQP was investigated and extended with multi-resolution and multi-orientation schemes in [29] to classify mammographic density. Their experimental results showed that the use of LQP based texture features improved the classification accuracy. In a recent study, Rampun et al. [30] tried local septenary patterns (LSP) method by using a seven-value encoding approach to further improve the classification performance. Their experimental results demonstrated that classification accuracy was slightly improved by LSP compared to LQP (80.5 ± 9.2 vs. 80.1 ± 10.5 on INBreast dataset).

Inspired by the success of texture analysis methods based on LBP and its variants, this paper investigates the usefulness of a richer texture descriptor by combining LBP and texture features obtained using multi-fractal analysis. Multi-fractal methods, due to their capability of enhancing image texture characteristics, have been used to process and interpret medical images [31,32,33]. Its applications in other image processing tasks also demonstrate that it can be an effective and promising tool for extracting texture features. To the best of our knowledge, multi-fractal analysis and its feature vectors have not been previously used to estimate breast density in mammograms. In this paper, we assume that a joint texture feature space consisting of LBP and multi-fractal features could possibly capture more effective and useful texture features related to breast density characteristics, thus improving the classification accuracy over methods using only one individual feature set. A FFDM dataset, INbreast, containing 409 mammogram images with the BI-RADS density labels annotated by radiologists as the ground truth is used in experiments to test the proposed methods. More details of our experimental approach are given in the following sections.

This paper is organized as follows. The next section describes a digital mammogram dataset used in experiments and the processing pipeline. Section 3 introduces two feature extraction methods: multi-fractal analysis and LBP. Section 4 discusses PCA and autoencoder model for feature selection, and Section 5 shows our experimental results and comparative analysis. Conclusions and some future work directions are given in Section 6.

## 2. Materials and Methods

### 2.1. Breast Density Classification

According to BI-RADS (4th edition) [6], breast density classification depends on the proportion of dense fibro-glandular tissue within the breast area in mammograms. The four categories are: (a) BI-RADS I (0–25% dense tissue, predominantly fat); (b) BI-RADS II (26–50% dense tissue, fat with some fibro-glandular tissue); (c) BI-RADS III (51–75% dense tissue, heterogeneously dense); and (d) BI-RADS IV (above 75% dense tissue, extremely dense). In many countries, radiologists estimate mammographic density using visual assessment, leading to the inter- and intra-readers variability [8].

### 2.2. Dataset

**Figure 1:**
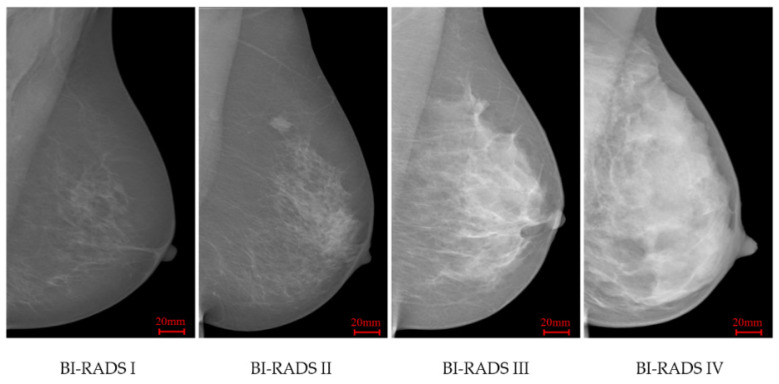
INbreast mammograms in four different breast density categories.

INbreast [34] is a FFDM database which contains 115 cases and 409 images including bilateral mediolateral oblique (MLO) and craniocaudal (CC) views. This database was acquired at the Breast Centre in CHSJ, Porto, under permission of both the Hospitals Ethics Committee and the National Committee of Data Protection. The images were acquired between April 2008 and July 2010; the acquisition equipment was the MammoNovation Siemens FFDM, with a solid-state detector of amorphous selenium, pixel size of 70 mm (microns), and 14-bit contrast resolution. The image matrix was 3328 × 4084 or 2560 × 3328 pixels, depending on the compression plate used in the acquisition (based on the breast size of the patient). This dataset offers carefully associated ground truth (GT) annotations by radiologists. For breast density, BI-RADS labels are available, including 136, 146, 99 and 28 images respectively in BI-RADS I to IV classes. Figure 1 illustrates four INbreast mammograms in different density categories.

### 2.3. Processing Stages

**Figure 2:**
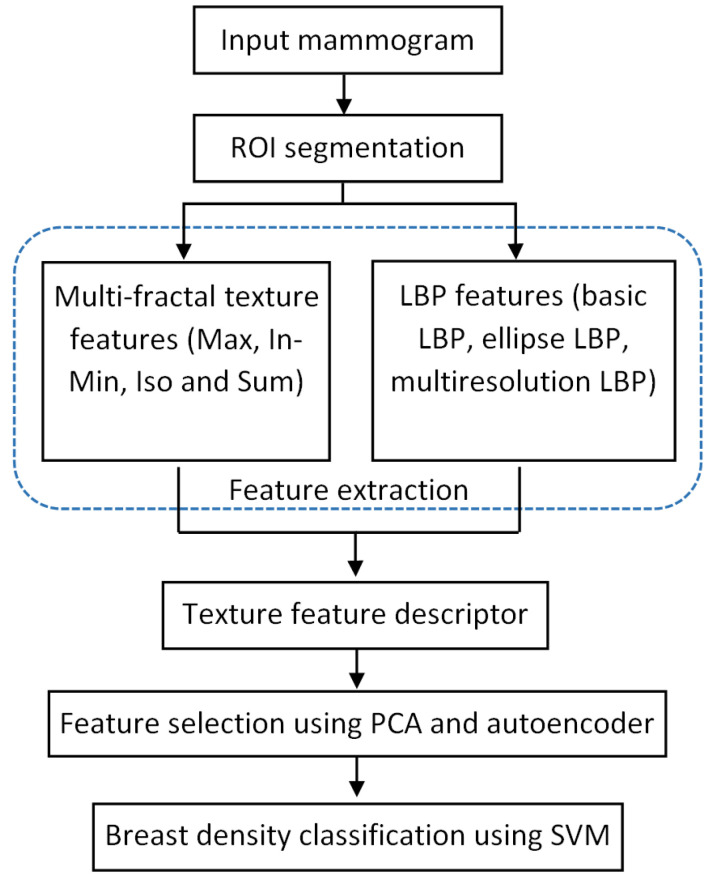
Processing steps for classifying breast density in mammograms.

Various stages of the processing pipeline are shown in Figure 2. After reading an input mammogram image, we first use a pre-processing step to segment a region of interest (ROI) which is the breast region in this work. The ROI segmentation results in a mask image which can be used in the feature extraction step, where we use texture features extracted from only the breast region areas (excluding the background area and pectoral muscle region). Multi-fractal analysis and local binary patterns are used to extract texture features from breast regions, constituting the texture descriptors. PCA and autoencoder network are used in the feature selection step to reduce the feature space. SVM is chosen as the classifier to predict the breast density label for each test mammogram image. These steps are detailed in the following sections.

### 2.4. Mammogram Pre-Processing

**Figure 3:**
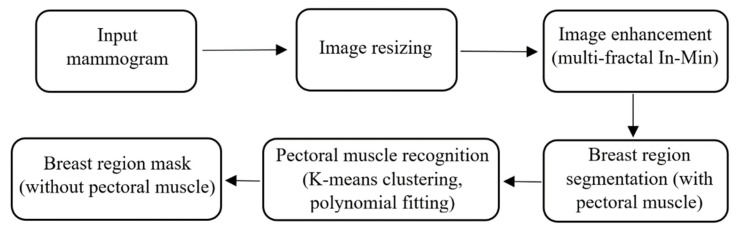
The pipeline of the pre-processing stage.

**Figure 4:**
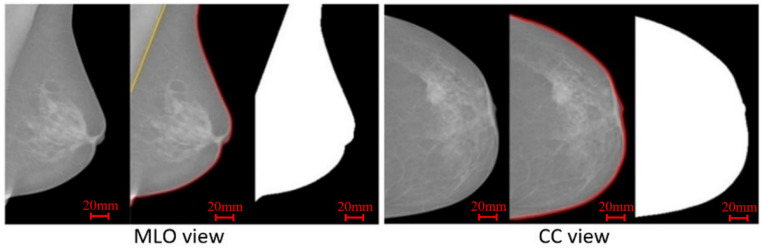
Two examples from the INbreast dataset, showing the breast region segmentation and mask images.

The main task performed in the pre-processing stage is the breast area segmentation. In MLO view mammograms, the pectoral muscle region is captured along with the breast region. However, the pectoral muscle represents a predominantly dense region which may easily affect breast density evaluation. Therefore, ROI segmentation is first applied to remove non-breast areas such as the background region and pectoral muscle. A pipeline for the pre-processing stage is shown in Figure 3. A multi-fractal method (introduced in Section 3.1 and Section 3.2) is used to enhance the contrast between image background and the breast tissue region. An intensity thresholding method and morphological operations [35] are used to separate the breast region from the background. A K-means algorithm and polynomial fitting approach [36] are employed to distinguish the pectoral muscle region from the breast region in MLO view mammograms. A median filter of 3 × 3 size is used to reduce noise. Finally, mask images are obtained, which are used to extract image features from only the region of interest (breast area) in the following steps. Figure 4 shows examples of segmenting the breast region from INbreast mammograms. A more detailed introduction of the methods used in this pre-processing stage can be found in the Supplementary Material (Figures S1 and S2). 

## 3. Feature Extraction

### 3.1. Multi-Fractal Analysis 

**Figure 5:**
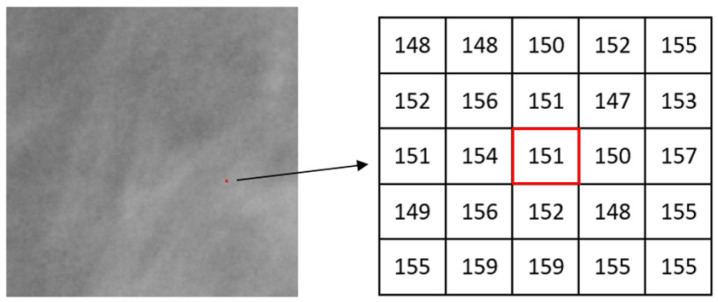
A local region (200 × 200 pixels) in one mammogram, and the central pixel *p*(151) and its neighbourhood.

**Figure 6:**
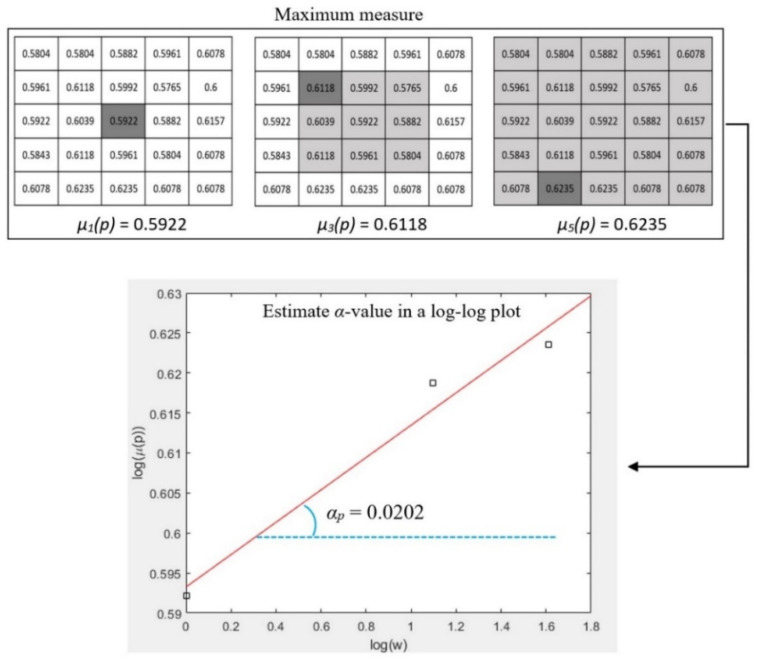
An example of estimating α value for the point *p* in Figure 5 using the maximum measure.

Multi-fractal analysis of image intensity variations computed using a set of local measures, can be used to describe image texture features for different classification tasks [31,32,33]. Let *µ_w_(p)* denote a multi-fractal measure function, where *p* is the central pixel in a square window of size *w* × *w*. Then, a local singularity coefficient, known as the Hölder exponent or *α*-value [37], can be calculated to reveal the variation of the selected *µ_w_(p)* function within the neighbourhoods of the pixel *p*.

(1) $$\mu_{w}\left(p\right)=Cw^{\alpha_{p}},\;w=2i+1,\;i=0,\;1,\;2,\ldots ,d$$

(2) $$log\left(\mu_{w}\left(p\right)\right)=\alpha_{p}log\left(w\right)+log\left(C\right)$$

where, *C* is an arbitrary constant and *d* is the total number of windows used in the computation of *α_p_*. The value of *α_p_* can be estimated from the slope of a linear regression line in a log-log plot where *log*(*µ_w_*(*p*)) is plotted against *log*(*w*). Commonly used multi-fractal measures for calculating *α* are outlined below:

(3) $$Maximum:\mu_{w}\left(p\right)=\underset{\left(k,l\in Ω\right)}{max}g\left(k,l\right)$$

(4) $$Inverse-minimum:\mu_{w}\left(p\right)=1-\underset{\left(k,l\right)\in Ω}{min}g\left(k,l\right)$$

(5) $$Summation:\mu_{w}\left(p\right)=\sum_{\left(k,l\right)\in Ω}g\left(k,\;l\right)$$

(6) $$Iso:\mu_{w}\left(p\right)=\#\{\left(k,l\right)|g\left(p\right)≅g\left(k,l\right),\left(k,l\right)\in Ω\}$$

where, *g*(*k, l*) represents the intensity value of a pixel at position (*k, l*); Ω denotes the set of all neighbourhood pixels of *p* in the window; *#* is the number of pixels in a set. Pixel intensity values are normalized into the range of [0, 1] when considering maximum and inverse-minimum measures. Such normalization enhances subtle image features in the domain of Hölder exponents due to the non-linear amplifying effect of the logarithmic function. A patch (a small image region) of one mammogram is shown in Figure 5 and one pixel p (marked in red colour) is chosen for illustrating the calculation of the α value. Figure 6 shows the measured values of *µ_w_*(*p*) by using maximum measure when the square window sizes of *w* are 1, 3, and 5 respectively. The *α* value is then estimated using the slope of the linear regression line in a log-log plot (Equation (2)).

### 3.2. Alpha Image and Texture Features 

**Figure 7:**
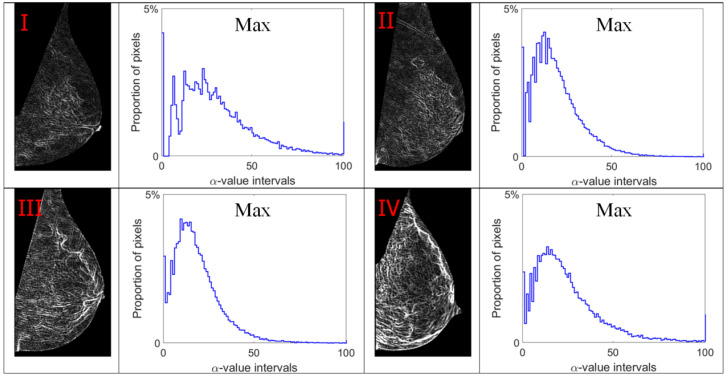
Alpha images and alpha histograms using the maximum measure. Up-left: BI-RADS (**I**) mammogram; up-right: BI-RADS (**II**) mammogram; down-left: BI-RADS (**III**) mammogram; down-right: BI-RADS (**IV**) mammogram.

**Figure 8:**
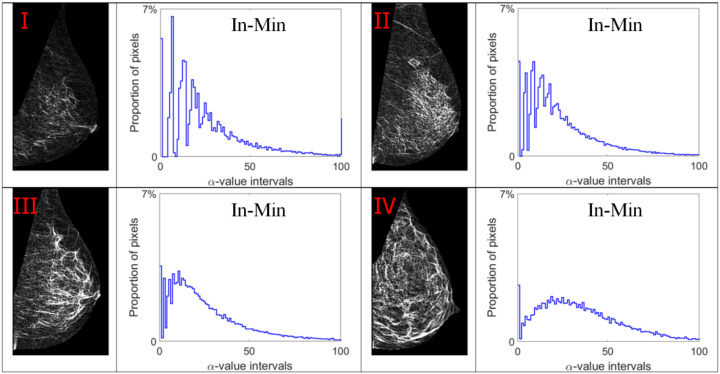
Alpha images and alpha histograms using the inverse-minimum measure. Up-left: BI-RADS (**I**) mammogram; up-right: BI-RADS (**II**) mammogram; down-left: BI-RADS (**III**) mammogram; down-right: BI-RADS (**IV**) mammogram.

**Figure 9:**
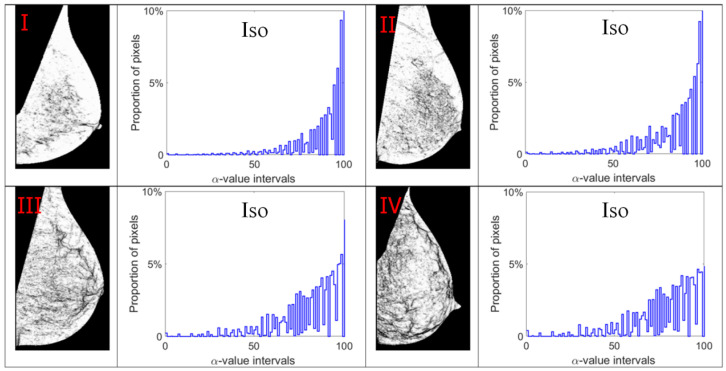
Alpha images and alpha histograms using the Iso measure. Up-left: BI-RADS (**I**) mammogram; up-right: BI-RADS (**II**) mammogram; down-left: BI-RADS (**III**) mammogram; down-right: BI-RADS (**IV**) mammogram.

**Figure 10:**
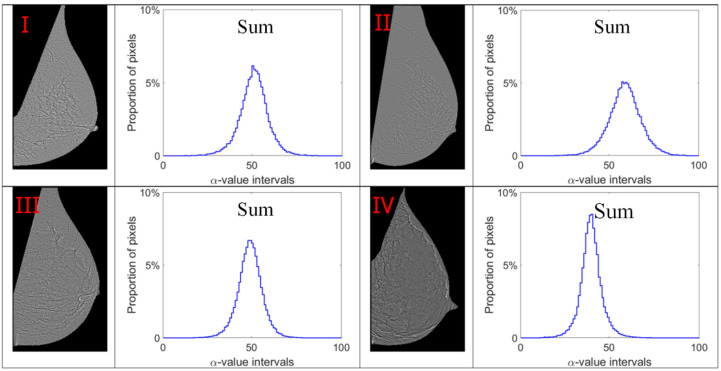
Alpha images and alpha histograms using the summation measure. Up-left: BI-RADS (**I**) mammogram; up-right: BI-RADS (**II**) mammogram; down-left: BI-RADS (**III**) mammogram; down-right: BI-RADS (**IV**) mammogram.

Alpha images (denoted as α-images) are obtained by replacing the intensity value at each position *p* by the *α_p_* value. In *α*-images, certain texture patterns have higher contrast compared to original images. The range of α values in an *α*-image is denoted by [*α_min_*, *α_max_*]. This range is subdivided into a set of bins, and pixels having the *α* values in the same bin are counted to obtain an *α*-histogram which can be used as one of the texture features [31,32]. In this study, the *α* value range in each mammogram image is divided into 100 bins which are used to obtain the corresponding *α*-histograms. As this work focuses on breast density classification, we extract texture features from only breast regions (pectoral muscle and background area excluded). Breast region mask images obtained in the pre-processing stage are used to control that the *α*-images and *α*-histograms reflect the image information in breast regions. In addition, since the area of breast region differs between different mammograms, we use the proportion of pixels in the region of interest to represent the height of bins in histograms instead of the pixel number, which makes the histogram based features uniform and comparable. Figure 7, Figure 8, Figure 9 and Figure 10 show *α*-images of mammograms in different breast density categories given earlier in Figure 1, and their *α*-histograms using four different multi-fractal measures.

### 3.3. Local Binary Patterns

Local binary pattern (LBP) proposed by [24] is a powerful feature descriptor used for texture analysis and classification. Due to its simplicity and robustness, several research works and applications use it to extract image features. For mammographic density classification, LBP and its variants have been applied and tested to improve the classification accuracy in [25,26,27,28,29,30]. A binary pattern is derived by comparing the intensity at each pixel with its neighbours and encoding the information in a *P*-bit integer value. Concretely, for each central pixel *c* with a grey level value *g_c_* in a specific window size, its LBP code is calculated by comparing the *g_c_* value with its neighbourhood pixels which is located at a distance *R* from *c*. If *g_c_* is higher than the neighbouring pixel *P_i_*, then the neighbour pixel is assigned a value 0, otherwise a value 1. Subsequently, a *P*-bit binary code is generated for the current pixel *c*. *LBP^P,R^* is used to denote this binary code and its calculation can be described as follows:

(7) $$LBP^{P,R}\left(x_{c},y_{c}\right)=\sum_{i=0}^{P-1}s\left(g_{i}^{P,R}-g_{c}\right)2^{i}\;$$

(8) $$s\left(x\right)=\{\begin{array}{c} 1,\;\;if\;x\geq 0\\ \;0,\;\;if\;x<0\; \end{array}$$

**Figure 11:**
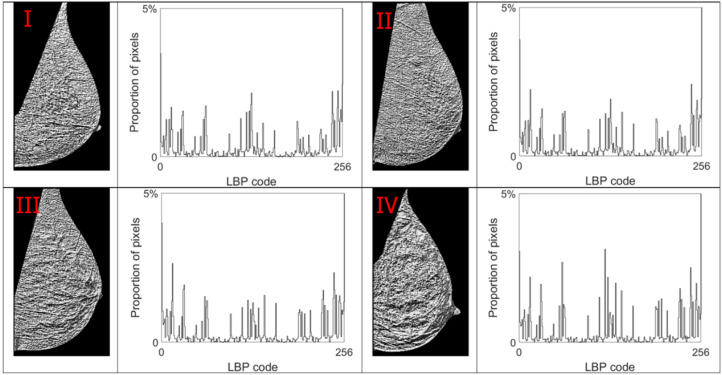
LBP code images and LBP histograms for mammograms in different density categories. Up-left: BI-RADS (**I**) mammogram; up-right: BI-RADS (**II**) mammogram; down-left: BI-RADS (**III**) mammogram; down-right: BI-RADS (**IV**) mammogram.

When *P* is set to 8, the total number of different binary patterns that can be generated using LBP is 256 (2^8^). Therefore, an LBP histogram containing 256 bins is obtained and used as a local texture descriptor. Figure 11 shows LBP images and their LBP histograms for mammograms in different density categories.

Larger neighbourhood areas represented by higher values of *R* provide more local image information and generate a longer texture feature vector. For example, when *P* = 8, setting *R* = 1 and *R* = 2 separately, two LBP histograms are obtained and concatenated, producing a 512-length feature vector. This descriptor is called a multi-resolution LBP (MLBP), and it contains more texture information and consequently has higher feature dimensionality compared to LBP.

In basic LBP, a circular neighbourhood with a radius of *R* is used for locating neighbouring pixels. As a variant of LBP, elliptical LBP (ELBP) is developed in [25], aiming to extract more local information from different directions. In ELBP, *R*_1_ and *R*_2_ denote the lengths of the semi-minor and semi-major axis of the elliptical image region, and the ELBP is calculated as follows:

(9) $$ELBP^{P,\;R_{1},\;R_{2}}\left(x_{c},y_{c}\right)=\sum_{i=0}^{P-1}s\left(g_{i}^{P,R_{1},\;R_{2}}-g_{c}\right)2^{i}\;$$

**Figure 12:**
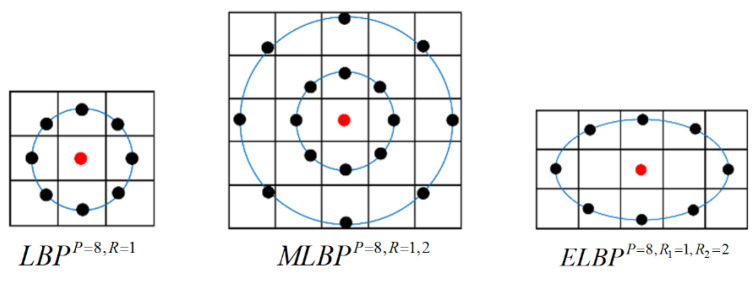
Illustrations of the basic LBP, MLBP and ELBP.

Figure 12 illustrates the configuration of image pixels used in the computation of the basic LBP and its variants discussed above, and they will be used and compared in this classification work.

### 3.4. Feature Descriptor with Concatenated Texture Features

As discussed above, multi-fractal features and LBP features are used as texture descriptors in this work to classify mammogram images. In addition to applying the two texture feature descriptors separately, we also create a novel feature descriptor by concatenating multi-fractal measure-based feature set with the LBP based feature set. By combining two different texture feature groups, we assume that more useful image information can be captured to reflect different tissue texture patterns related to breast density characteristics, thus improving the classification performance. In our experiments (Section 4), the four different multi-fractal measures (i.e., Max, In-Min, Iso, Sum) discussed in Section 3.2 are concatenated with LBP based texture features separately with the aim of obtaining a higher classification accuracy. 

## 4. Feature Selection

The concatenation of several texture feature vectors could result in high feature dimensionality and a high level of feature redundancy. In this section, we consider using PCA and the autoencoder network to select the optimum subset of texture features by removing redundant features which possibly do not relate to the breast density characteristics well.

### 4.1. Principal Components Analysis

Principal components analysis (PCA) is used for efficient coding of various biological signals [38]. It is a well-known optimal linear scheme for dimension reduction in data analysis, which retains maximal variance in the data set, while improving algorithm performance and saving processing time.

In the proposed method, *X* is used to denote the input feature set. *X* is an *M* × *N* matrix which has *N* dimensional features and *M* elements in each dimensionality. We use *X^i^* to refer to the entire set of elements in the *i*th dimension and *X_j_^i^* to refer to the *j*th element in this set. The covariance between the first two dimensions *X*^1^ and *X*^2^ is computed as below.

(10) $$cov\left(X^{1},X^{2}\right)=\frac{\sum_{j=1}^{M}\left(X_{j}^{1}-\bar{X^{1}}\right)\left(X_{j}^{2}-\bar{X^{2}}\right)}{M-1}$$

where, $\bar{X^{1}}$ and $\bar{X^{2}}$ denote means of the set of *X*^1^ and *X*^2^ respectively. After computing all the possible covariance values between different dimensions, a covariance matrix CM can be obtained as follows:

(11) $$CM=\left\{\begin{array}{ccc} cov\left(X^{1},X^{1}\right) & \ldots & cov\left(X^{1},X^{N}\right)\\ \vdots & \ddots & \vdots \\ cov\left(X^{N},X^{1}\right) & \ldots & cov\left(X^{N},X^{N}\right) \end{array}\right\}$$

Eigenvalues and eigenvectors of the covariance matrix are calculated subsequently, and eigenvectors are sorted in descending order according to the eigenvalues. A matrix *V* can be constructed with these eigenvectors in columns. The final feature set *X*′ can be derived from *X* and the matrix *V* as follows:

(12) $$X^{\prime }=V^{T}\times X^{T}$$

In PCA, an assumption made for feature selection is that most information of the input feature set is contained in the subspace spanned by the first *n* principal axes, where *n* < *N* in an *N*-dimensional feature space. Therefore, each original feature vector can be represented by its principal component vector with the dimensionality of *n*.

### 4.2. Autoencoder Network

Autoencoder network [39] is a feed-forward neural network with more than one hidden layer, which attempts to reconstruct input data at the output layer. The output layer is usually of the same size as the input layer and the network architecture represents an hour-glass shape. As the size of the hidden layer in an autoencoder neural network is smaller than the input layer, the high-dimensional input data can be reduced to narrower code space when using more hidden layers. Therefore, in addition to image reconstruction and compression [40], an autoencoder is also used to reduce feature dimensionality. Generally, an autoencoder network consists of two components, namely “encoder” and “decoder”. By reducing the hidden layer size, the encoder part is forced to learn important features of the input data, and the decoder part reconstructs the original data from the generated feature code. Once the training phase is over, the decoder part is discarded and the encoder is used to transform a data sample to a feature subspace.

**Figure 13:**
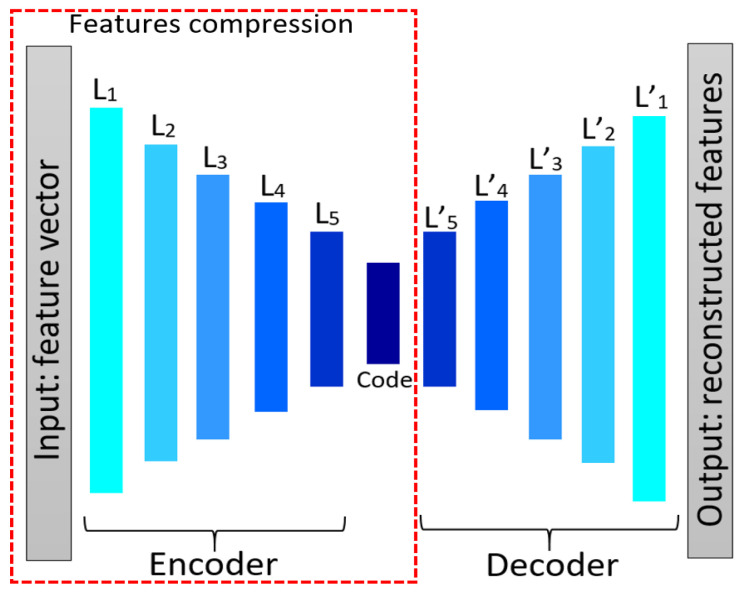
The autoencoder architecture for reducing feature numbers.

We use an autoencoder to reduce the length of the feature vector. Therefore, a feature vector of size (1 × *n*) is input into the autoencoder network. Figure 13 shows an autoencoder model containing 11 hidden layers, in which the input layer receives and processes the initial texture features. Fully connected (FC) layers are used as hidden layers and rectified linear unit (ReLU) is used as the activation function in each hidden layer except the last layer which uses a sigmoid function. Binary cross entropy is employed as a loss function.

### 4.3. Classification

SVM is used in this study for training the classification model and producing classification results on test images. Since this work aims to classify mammograms into multiple density categories, a multiclass SVM which is implemented by a one against all (OAA) method is used in this model. To obtain the optimal classification results, three commonly used kernels [41], RBF, Poly, and Sigmoid, are tested in this work. A grid-searching method is used to find the best combinations of parameters (*gamma*, *C*, and *degree*).

## 5. Experiments and Result Analysis

In our experiments, a five-fold cross validation is performed on INbreast dataset using the proposed texture descriptors and the SVM classifier. Table 1 lists the important parameters of the methods used and their selected values or value ranges.

### 5.1. Classification Results Using Multi-Fractal Features 

We first test and compare the efficacy of multi-fractal feature descriptors for classifying breast density. As discussed in Section 3.2, four different multi-fractal measures are used to obtain the *α*-histograms which contain 100 bins in each histogram. The *α*-histogram is used as the texture feature descriptor to represent the breast density related features. One out of four BI-RADS density labels are predicted for each test mammogram by the SVM classifier. The classification results are shown in confusion matrices in Table 2 for each of the four different multi-fractal measures. From the confusion matrices we can see that the classification results based on different feature descriptors differ significantly. The Iso based feature descriptor produces the highest classification accuracy of 73.8%, while an accuracy of 63.3% is obtained using features based on the Max measure. The other two multi-fractal measures, In-Min and Sum, yield lower accuracy results of 59.7% and 55.3% respectively.

### 5.2. Classification Results Using LBP Features 

Three LBP based texture feature descriptors, LBP, ELBP, and MLBP, are tested under the same experimental setting. As introduced in Section 3.3, the LBP or ELBP based histogram contains 256 bins, while the MLBP calculates LBP codes from two different neighbourhoods, containing a total of 512 bins. Classification results in Table 3 show that the MLBP feature descriptor produces a slightly higher classification accuracy (73.3%) than the other two descriptors. The accuracy of 72.1% is obtained using ELBP and 70.9% is observed using LBP. The MLBP descriptor contains much more image features extracted from larger local areas, and as expected, produces a better classification performance. Therefore, MLBP is selected to be concatenated with different multi-fractal feature sets in next section, constituting new feature descriptors.

### 5.3. Classification Results Using Cascaded Features 

In this section, we discuss the possibility of augmenting the feature space by concatenating multi-fractal texture features and the MLBP features. Each multi-fractal measure (Max, In-Min, Iso, and Sum) based feature set cascades the MLBP feature set, generating a 612-bin feature descriptor. To reduce the feature dimensionality by removing irrelevant texture features, PCA is used to optimize the feature space before using the feature descriptor to classify mammograms. The final classification results are shown in Table 4. The overall classification performance is improved by more than 10%, compared to the results obtained using individual multi-fractal feature descriptors or the LBP based features. The best classification accuracy of 84.6% is obtained using the Iso+MLBP feature descriptor. The Max+MLBP feature set also gives a high accuracy of 81.4%. By combining MLBP features, the In-Min and Sum based descriptors also show improved accuracy levels of 76.8% and 68.9% respectively. This indicates that the feature combination and the use of PCA contribute to improving the representation ability and discriminating power of texture features for distinguishing breast density related characteristics in mammograms. 

### 5.4. Effect of Feature Selection

This section investigates the effect of feature selection using PCA and autoencoder network on classification accuracy. As discussed in previous sections, the cascaded texture feature set (multi-fractal features + MLBP) occupies a large feature space which may contain redundant image features, and need to be optimized before using it in the classification algorithm. 

**Figure 14:**
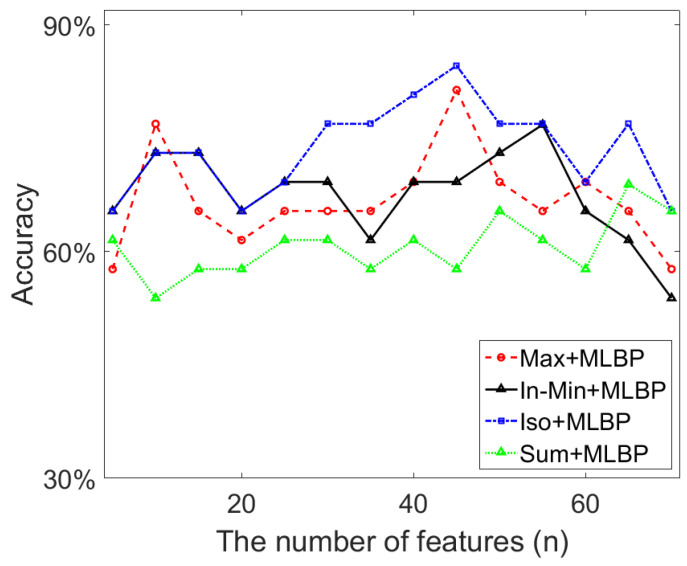
The classification accuracy using the reduced texture features by PCA.

PCA re-computes the initial feature set and outputs new features in decreasing order, which can be used to select the optimum *n* features for the classification work. Figure 14 shows the top 70 features after applying PCA, in which range the best classification results shown in Table 4 are obtained when using the cascaded feature descriptors. In Figure 14, we can see that the highest classification accuracy (84.6%) based on the Iso+MLBP descriptor is obtained using the top 45 (i.e., *n* = 45) features. The Max+MLBP descriptor also uses *n* = 45 features to reach its best classification performance. For the In-Min and Sum based descriptors, 55 and 65 features respectively are used to achieve their best classification results. As the initial feature set contains 612 features, over 90% feature space is removed by PCA, which contributes to a significant reduction in feature dimensionality and the improvement of classification accuracy. 

We also use the autoencoder network to select texture features for the cascaded feature descriptors. Different number of hidden layers in the autoencoder structure are tested using values derived from the set {5,7,9,11,13, and 15} and classification results based on the Iso+MLBP descriptor are shown in Table 5. An 11-layer architecture with the code layer size of 64 produces the highest classification result (80.7%). The results indicate that the autoencoder network can optimize the feature space for the cascaded feature descriptors and obtain a desirable classification accuracy. However, the classification performance does not surpass the accuracy (80.7% vs. 84.6%) by using PCA.

### 5.5. Results Comparison and Discussion

In this section, we further compare and discuss the experimental results presented in Section 5.1, Section 5.2 and Section 5.3 using different classification metrics such as accuracy (CA), AUCROC, Kappa coefficient and F1 score. In addition, statistical hypothesis test is conducted. The *t*-test with a significance level of 0.05 is conducted between the Iso+MLBP and every other method to calculate *p*-value, which shows the statistical difference between them. Table 6 shows that the Iso+MLBP joint feature descriptor outperforms other approaches, not only producing the highest AUC (95.3 ± 3.1) but also obtaining higher Kappa (0.79), and F1 score (0.85). In the t-test, all other methods present low *p*-values (<0.05), which means the difference of classification performance is statistically significant.

Based on the classification results shown in Table 2, Table 3, Table 4 and Table 6, we have the following findings:

(1) For the LBP and its variants, we can see that the use of a different neighbourhood topologies (i.e., elliptical vs. circular) can improve the classification performance, which is consistent with the conclusion in [25]: the extracted anisotropic texture information have the potential in distinguishing objects. However, the MLBP which collects texture information from two different neighbourhood areas makes the improvement more evident. This indicates that for the breast density classification which is a very challenging task due to the heterogeneous texture patterns of breast tissue, capturing more (richer) texture features from different local regions can lead to the improvement of the classification performance.
(2) The results in Table 2 lead to the following observations regarding multi-fractal features. The Iso measure produces a better classification result than the other measures. The reason for this can be ascribed to fact that the Max and In-Min measures consider only one pixel information (the maximum or the minimum intensity value) when computing the singularity coefficient (i.e., *α*-value), and fails to collect the image information from the other pixels within the local region.
(3) From the results in Table 4 and Table 6, we can see that the use of the combined feature descriptor improves the classification accuracy significantly, which also indicates that the texture features extracted from the MLBP and multi-fractal methods are different. The feature sets collected by the two different methods can be considered as complementary to each other.
(4) As shown in Section 5.4, the combination of different texture features produce a bigger feature space which contains redundant features that do not help distinguish the breast density related characteristics between different categories. Results in Figure 14 show that the classification accuracy can even be lower than using the individual feature set if the concatenated features are not selected properly, which demonstrate the importance of removing the redundant features and the necessity of using the feature selection scheme.
(5) Even though BI-RADS uses four density categories, sometimes, breast density is discussed with binary labels of low density (fatty and sparsely dense, or BI-RADS I and II) and high density (heterogeneously and extremely dense, or BI-RADS III and IV) [7]. We conduct the binary classification using the Iso+MLBP descriptor which produces the best four-category classification results in our experiments. Table 7 shows the binary classification results. Although the texture features extracted by the multi-fractal Iso method and the MLBP provide desirable binary classification accuracies (89.2% and 91.9%), the joint feature descriptor Iso+MLBP shows a more powerful representation capability for image features, with a higher classification performance of 92.9% obtained. These observations are consistent with the results obtained in four-category classification work and demonstrates the robustness of the proposed feature descriptor.

A recent study [29] applied a local quinary pattern (LQP) method to extract texture features using different neighbourhood topologies for the same classification task. Their results indicate that the ellipse topology based LQP gives the best accuracy of 82.02% when using over 200 image features to test 206 images in the INbreast dataset (only MLO view images used). By contrast, our proposed method is tested on the whole INbreast dataset (409 images) and attains the accuracy of 84.6% using only 45 features. Table 8 shows the comparison of classification performance by using LQP, LBP and multi-fractal feature descriptors. 

## 6. Conclusions and Future Work

In this paper, a comprehensive study towards the multi-fractal analysis is conducted to extract texture features in mammogram images for classifying BI-RADS density. Four different multi-fractal measures are used to capture breast density related image features, and LBP and its variants are employed to extract more features. Novel texture feature descriptors concatenating multi-fractal features and MLBP features are proposed to integrate rich image features based on different feature extraction methods. PCA and autoencoder network are used in this work to optimize the feature space by removing redundant features. The proposed method is tested using a FFDM dataset, INBreast, with 409 mammogram images. Experimental results show that the cascaded feature descriptor based on multi-fractal analysis and LBP obtains the higher classification accuracy than using individual feature set. In future work, other image feature descriptors such as LQP will be considered together with multi-fractal features. In addition, different mammogram datasets will be used in experiments to demonstrate the robustness of the proposed method.

## Figures and Tables

**Table 1:** Methods, parameters and their value ranges used in experimental analysis.

Method	Related Parameters
Multi-fractal analysis	Window edge size: *w* (for calculating alpha values), *w* = {1, 3, 5} Local measures: Max, In-Min, Iso, Sum Feature number *n* = 100
LBP	1. (*R* = 2, *P* = 8)
MLBP	1. (*R*_1_ = 2, *R*_2_ = 4, *P* = 8)
ELBP	1. (*R*_1_ = 1, *P*_1_ = 8), (*R*_2_ = 4, *P*_2_ = 8)
Autoencoder network	The number of hidden layers tested: {5, 7, 9, 11, 13, and 15} Feature number *n* = {16, 32, 64, 128}
SVM classifier	For grid-searching: Kernel candidates: RBF/Poly/Sigmoid *γ* searching range: [10^−4^, 10^3^] *C* searching range: [10^−3^, 10^4^] Degree (only Poly kernel) searching range: {1, 2, 3, 4, 5, 6}

**Table 2:** Confusion matrices of classification results using multi-fractal feature descriptors.

BI-RADS		Predicted (Max)				Accuracy = 63.3%	BI-RADS		Predicted (In-Min)				Accuracy = 59.7%
		I	II	III	IV	Recall			I	II	III	IV	Recall
Actual	I	113	8	11	4	83%	Actual	I	98	16	18	4	72%
	II	49	51	44	2	35%		II	38	65	39	4	45%
	III	20	1	73	5	74%		III	18	8	65	8	66%
	IV	2	0	4	22	79%		IV	5	0	7	16	57%
BI-RADS		Predicted (Iso)				Accuracy = 73.8%	BI-RADS		Predicted (Sum)				Accuracy = 55.3%
		I	II	III	IV	Recall			I	II	III	IV	Recall
Actual	I	119	9	5	3	88%	Actual	I	88	21	25	2	65%
	II	24	91	26	5	62%		II	41	69	36	0	47%
	III	15	5	72	7	73%		III	21	10	59	9	60%
	IV	0	1	7	20	71%		IV	1	5	12	10	36%

**Table 3:** Confusion matrices of classification results using LBP based feature descriptors.

BI-RADS		Predicted (LBP)				Accuracy = 70.9%	BI-RADS		Predicted (ELBP)				Accuracy = 72.1%
		I	II	III	IV	Recall			I	II	III	IV	Recall
Actual	I	127	0	9	0	93%	Actual	I	125	5	6	0	92%
	II	48	69	29	0	47%		II	49	68	29	0	47%
	III	16	3	80	0	81%		III	11	3	83	2	84%
	IV	0	0	14	14	50%		IV	0	0	9	19	68%
BI-RADS		Predicted (MLBP)				Accuracy = 73.3%							
Actual		I	II	III	IV	Recall							
	I	129	3	4	0	95%							
	II	50	72	24	0	49%							
	III	11	2	85	1	86%							
	IV	1	0	13	14	50%							

**Table 4:** Confusion matrices of classification results using cascaded texture feature descriptors.

BI-RADS		Predicted (Max + MLBP)				Accuracy = 81.4%	BI-RADS		Predicted (In-Min + MLBP)				Accuracy = 76.8%
		I	II	III	IV	Recall			I	II	III	IV	Recall
Actual	I	130	2	3	1	96%	Actual	I	129	3	3	1	95%
	II	36	95	15	0	65%		II	32	88	25	1	60%
	III	10	2	84	3	85%		III	14	4	73	8	74%
	IV	1	0	3	24	86%		IV	1	0	3	24	86%
BI-RADS		Predicted (Iso + MLBP)				Accuracy = 84.6%	BI-RADS		Predicted (In-Min + MLBP)				Accuracy = 68.9%
		I	II	III	IV	Recall			I	II	III	IV	Recall
Actual	I	128	5	2	1	94%	Actual	I	107	10	14	5	79%
	II	18	108	19	1	74%		II	40	76	30	0	52%
	III	8	1	84	6	85%		III	11	4	76	8	77%
	IV	0	0	2	26	93%		IV	1	1	3	23	82%

**Table 5:** The classification accuracy using Iso+MLBP feature descriptor and different structures of the autoencoder network.

The Number of Hidden Layers	Feature Number	Classification Accuracy
5	128	72.9%
7	128	75.2%
9	64	77.2%
11	64	80.7%
13	32	76.2%
15	16	69.1%

**Table 6:** The comparison of classification performance between different feature descriptors, with the best classification performance highlighted in bold.

	CA (%)	AUC (%)	Kappa	F1	*p*-Value
LBP	70.9	85.6 ± 3.6	0.58	0.72	<10^−4^
ELBP	72.1	86.9 ± 2.8	0.59	0.73	<10^−4^
MLBP	73.3	87.2 ± 2.9	0.60	0.74	0.015
Multi-fractal (Max)	63.3	80.1 ± 4.4	0.41	0.63	0.001
Multi-fractal (In-Min)	59.7	78.3 ± 3.5	0.34	0.60	<10^−4^
Multi-fractal (Iso)	73.8	87.4 ± 4.1	0.60	0.74	0.002
Multi-fractal (Sum)	55.3	77.1 ± 4.5	0.31	0.55	0.001
Iso+MLBP	**84.6**	**95.3 ± 3.1**	**0.79**	**0.85**	−

**Table 7:** Confusion matrices of the binary density categories classification results using the proposed feature descriptors.

Binary Categories		Predicted (MLBP)		Accuracy = 91.9%	Binary Categories		Predicted (Iso)		Accuracy = 89.2%
		Low Density	High Density	Recall			Low Density	High Density	Recall
Actual	Low density	259	23	92%	Actual	Low density	253	29	90%
	High density	10	117	92%		High density	15	112	88%
Binary categories		Predicted (Iso + MLBP)		Accuracy = 92.9%					
		Low Density	High Density	Recall					
Actual	Low density	262	20	93%					
	High density	9	118	93%					

**Table 8:** The classification accuracy comparison using different feature descriptors.

Image Feature	The Number of Features	The Number of Test Images	Classification Accuracy (%)
LQP(Ellipse) [29]	Over 200	206	82.02%
LQP(Circle) [29]	Around 100	206	Under 80%
LBP	256	409	70.9%
ELBP	256	409	72.1%
MLBP	512	409	73.3%
Multi-fractal (Max)	100	409	63.3%
Multi-fractal (In-Min)	100	409	59.7%
Multi-fractal (Iso)	100	409	73.8%
Multi-fractal (Sum)	100	409	55.3%
Max+MLBP	45	409	81.4%
In-Min+MLBP	55	409	76.8%
Iso+MLBP	45	409	**84.6%**
Sum+MLBP	65	409	68.9%

## Data Availability

Not applicable.

## Supplementary Materials

The following are available online at https://www.mdpi.com/article/10.3390/jimaging7100205/s1, Figure S1: Segmentation of breast region. (**a**) Input mammogram; (**b**) clustering operation based on the alpha image; (**c**) rough contour of the breast region; (**d**) smoothing breast contour; (**e**) finding pectoral muscle area; (**f**) breast mask image without pectoral muscle region; Figure S2: **.** Challenging cases with inaccurate mask images generated and their adjusted mask images based on manual operations. References [42,43,44] are cited in the Supplementary Materials.
