# Supplementary material for: Novel Texture Feature Descriptors Based on Multi-Fractal Analysis and LBP for Classifying Breast Density in Mammograms
Source: J Imaging. 2021 Oct 6;7(10):205. doi: 10.3390/jimaging7100205 (PMC8540831; doi:10.3390/jimaging7100205)
Supplement: Supplementary file 1 [file jimaging-07-00205-s001.zip › jimaging-1379255-supplementary.pdf]

Supplementary material

A. Introduction of the Pre-processing Stage

A brief introduction of the pre-processing work for segmenting ROI in mammograms and its pipeline (Figure 3) are given in Section 2.4. This supplementary material introduces more details on the methods used in the pre-processing stage.

Image resizing: A scale factor (s) was used to study the variations in classification accuracy with respect to changes in image size in our previous work [42], and experimental results showed that when the INbreast mammogram image is resized to smaller size ($s = 1/8$, the resized image is 1/8 times the size of original image), better classification results were obtained. Therefore, in this step, based on the findings in [42], we resized the INbreast mammogram images, from 3328 × 4084 or 2560 × 3328 pixels to 416 × 510 or 320 × 416 pixels. In addition, the image grey level is adjusted from 14bits to 8bits.

Image enhancement and breast region segmentation: The input mammogram is processed using multi-fractal In-Min method (introduced in Section 3.1) to generate the corresponding alpha-image (Figure S1b), for enhancing the image contrast. The alpha image is divided into 10 clusters by k-means method, and the cluster with the lowest mean is removed as it represents the background. Morphological opening with a disk of radius of five is applied to the image in order to remove any small objects and retain only the largest image segment that represents the breast. Filling of small holes is performed and an averaging filter is applied to obtain a smoother border of the breast. Following this step, a breast region mask with pectoral muscle is obtained (Figure S1d).

Pectoral muscle recognition: Within the breast region segmented using the mask image, intensity value-based image clustering is performed using the k-means algorithm. For this, the method used in [36] is implemented. Three different numbers of clusters are applied: in the first case, the image is divided into two, in the second it is divided into three, and in the third, it is divided into four clusters. In every image, the cluster with the highest mean value is kept. Finally, the top left corner object (for the left side breast) is assumed to represent the pectoral muscle (Figure S1e)). Polynomial fitting is used with the sampled points along with the pectoral muscle borderline to obtain a smooth contour (Figure S1f).

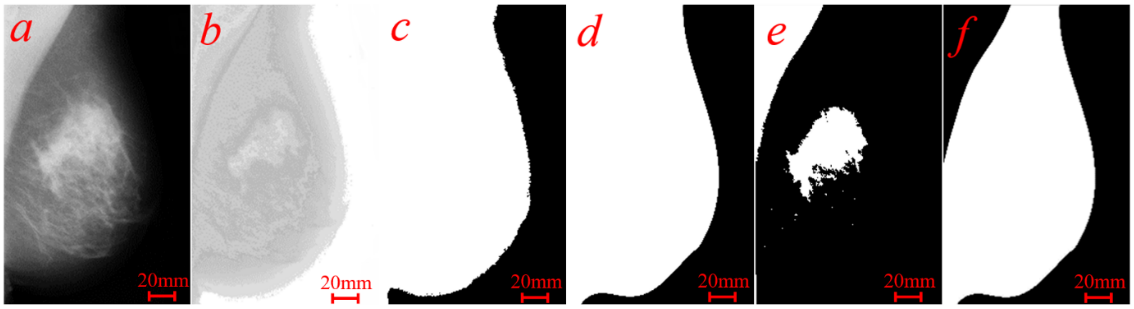

Figure S1. Segmentation of breast region. (**a**) Input mammogram; (**b**) clustering operation based on the alpha image; (**c**) rough contour of the breast region; (**d**) smoothing breast contour; (**e**) finding pectoral muscle area; (**f**) breast mask image without pectoral muscle region.

However, the pectoral muscle removal from the breast region area can be a challenging work for some difficult cases. Related works address this problem as a separate research topic [43,44] with different approaches proposed, which are beyond the scope of this paper.

Some mammograms in INbreast dataset present blurred boundary lines between the pectoral muscle area and the breast region, which result in inaccurate mask images. We found around 80 mammogram images (approximately 20%) in INbreast with poorly delineated boundaries, and the corresponding mask images were manually corrected with the help of a breast imaging radiologist. Figure S2 shows some examples of the challenging cases and their mask images before and after using manual operations.

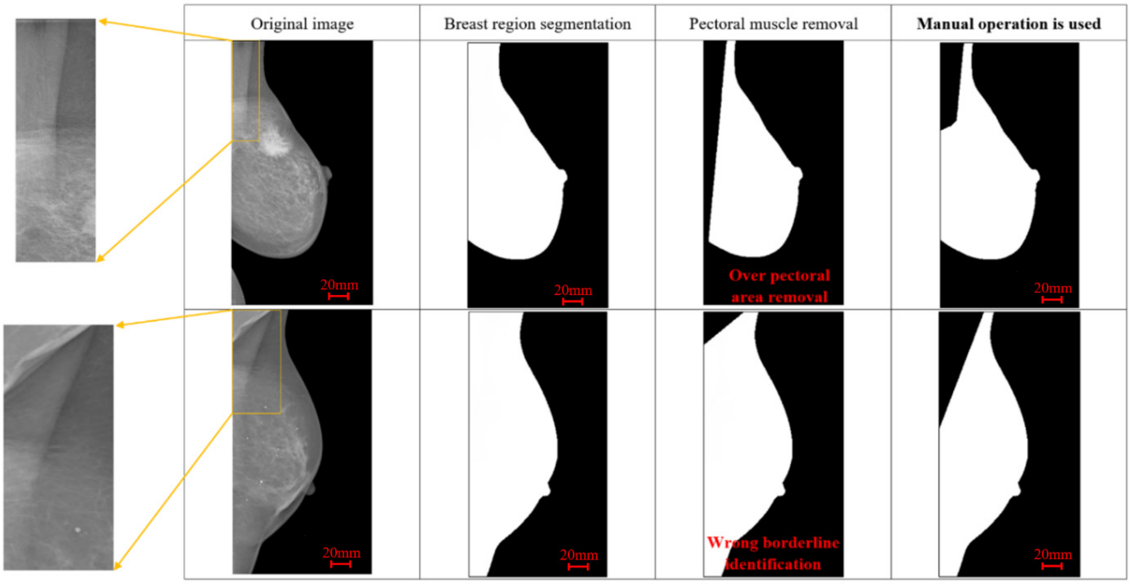

Figure S2. Challenging cases with inaccurate mask images generated and their adjusted mask images based on manual operations.

References

42. Li, H.; Mukundan, R.; Boyd, S. Robust Texture Features for Breast Density Classification in Mammograms. In Proceedings of the 16th International Conference on Control, Automation, Robotics and Vision (ICARCV), Shenzhen, China, 13–15 December 2020; pp. 454–459, https://doi.org/10.1109/ICARCV50220.2020.9305431.
43. Sreedevi, S.; Sherly, E. A Novel Approach for Removal of Pectoral Muscles in Digital Mammogram. *Procedia Comput. Sci.* **2015**, *46*, 1724–1731, https://doi.org/10.1016/j.procs.2015.02.117.
44. Rampun, A.; López-Linares, K.; Morrow, P.J.; Scotney, B.W.; Wang, H.; Ocaña, I.G.; Maclair, G.; Zwiggelaar, R.; Ballester, M.A.G.; Macía, I. Breast pectoral muscle segmentation in mammograms using a modified holistically-nested edge detection network. *Med Image Anal.* **2019**, *57*, 1–17, https://doi.org/10.1016/j.media.2019.06.007.
